# Characteristics of Dimensional Psychopathology in Suicidal Patients With Major Psychiatric Disorders and Its Association With the Length of Hospital Stay: Algorithm Validation Study

**DOI:** 10.2196/30827

**Published:** 2021-09-03

**Authors:** Dong Yun Lee, Jimyung Park, Jai Sung Noh, Hyun Woong Roh, Jae Ho Ha, Eun Young Lee, Sang Joon Son, Rae Woong Park

**Affiliations:** 1 Department of Biomedical Informatics Ajou University School of Medicine Suwon Republic of Korea; 2 Department of Biomedical Sciences Ajou University Graduate School of Medicine Suwon Republic of Korea; 3 Department of Psychiatry Ajou University School of Medicine Suwon Republic of Korea; 4 Office of Biostatistics, Medical Research Collaborating Center Ajou Research Institute for Innovative Medicine, Ajou University Medical Center Suwon Republic of Korea

**Keywords:** suicide, computed phenotype, natural language processing, research domain criteria, electronic health record

## Abstract

**Background:**

Suicide has emerged as a serious concern for public health; however, only few studies have revealed the differences between major psychiatric disorders and suicide. Recent studies have attempted to quantify research domain criteria (RDoC) into numeric scores to systematically use them in computerized methods. The RDoC scores were used to reveal the characteristics of suicide and its association with major psychiatric disorders.

**Objective:**

We intended to investigate the differences in the dimensional psychopathology among hospitalized suicidal patients and the association between the dimensional psychopathology of psychiatric disorders and length of hospital stay.

**Methods:**

This retrospective study enrolled hospitalized suicidal patients diagnosed with major psychiatric disorders (depression, schizophrenia, and bipolar disorder) between January 2010 and December 2020 at a tertiary hospital in South Korea. The RDoC scores were calculated using the patients’ admission notes. To measure the differences between psychiatric disorder cohorts, analysis of variance and the Cochran Q test were conducted and post hoc analysis for RDoC domains was performed with the independent two-sample *t* test. A linear regression model was used to analyze the association between the RDoC scores and sociodemographic features and comorbidity index. To estimate the association between the RDoC scores and length of hospital stay, multiple logistic regression models were applied to each psychiatric disorder group.

**Results:**

We retrieved 732 admissions for 571 patients (465 with depression, 73 with schizophrenia, and 33 with bipolar disorder). We found significant differences in the dimensional psychopathology according to the psychiatric disorders. The patient group with depression showed the highest negative RDoC domain scores. In the cognitive and social RDoC domains, the groups with schizophrenia and bipolar disorder scored higher than the group with depression. In the arousal RDoC domain, the depression and bipolar disorder groups scored higher than the group with schizophrenia. We identified significant associations between the RDoC scores and length of stay for the depression and bipolar disorder groups. The odds ratios (ORs) of the length of stay were increased because of the higher negative RDoC domain scores in the group with depression (OR 1.058, 95% CI 1.006-1.114) and decreased by higher arousal RDoC domain scores in the group with bipolar disorder (OR 0.537, 95% CI 0.285-0.815).

**Conclusions:**

This study showed the association between the dimensional psychopathology of major psychiatric disorders related to suicide and the length of hospital stay and identified differences in the dimensional psychopathology of major psychiatric disorders. This may provide new perspectives for understanding suicidal patients.

## Introduction

### Background

The World Health Organization states that nearly 800,000 people die each year from suicide, one every 40 seconds [1]. Most patients who committed suicide had psychiatric disorders [2]. Among psychiatric disorders, schizophrenia and affective disorders demonstrate the highest risk for suicide [3], but few studies have examined the differences in the suicide-related features of psychiatric disorders [4].

Suicide attempts vary depending on the method, intent, and medical severity of the aftereffects [5]. The length of hospital stay is especially related to the outcome of a patient hospitalized for suicide attempts [3] with some studies stating that patients with longer admissions are at greater risk of postdischarge suicide [6]. Understanding the psychiatric features of patients who stay longer in the hospital might help reduce the length of stay and perhaps their postdischarge outcomes [7]. Several studies have explored the factors associated with the length of stay in suicidal patients, but the results have been inconsistent [8].

Meanwhile, the diagnosis of psychiatric patients so far has relied on categorical diagnostic systems. As the limitations of categorical diagnostic systems became increasingly apparent, the research domain criteria (RDoC) was introduced as an alternate nosology by the National Institute of Mental Health (NIMH) [9]. Natural language processing (NLP) was introduced as one of the ways to use RDoC, and hospital readmission could be predicted with RDoC domains extracted by NLP [10]. Thus, NLP can be used effectively to evaluate psychiatric notes as RDoC domains [11].

### Objectives

In this study, we aimed to explore the differences in the RDoC domains extracted by NLP among patients with depression, schizophrenia, and bipolar disorder who were hospitalized for suicide attempts. We sought to determine whether narrative clinical notes could identify suicide-related features of each disorder. We also investigated how these domains were associated with the length of hospital stay and compared them for each disorder.

## Methods

### Data Collection

Clinical and sociodemographic data were extracted from the electronic health records of patients in the psychiatry inpatient unit at Ajou University Hospital in South Korea between 2010 and 2020. All patients received a Diagnostic and Statistical Manual of Mental Disorders (DSM)-IV-TR or DSM-5 diagnosis from a trained psychiatrist [12,13]. Clinical data included diagnosis (ie, depression, schizophrenia, and bipolar disorder) at admission and chief complaints at admission such as suicide attempts, suicide planning, and suicidal ideations. Sociodemographic data included the age, sex, length of stay, past medical history, and Charlson Comorbidity Index (CCI) score. Admission notes on the patients were extracted for estimating the RDoC scores by NLP. The data were encoded using the Observational Medical Outcomes Partnership (OMOP) common data model (CDM) (version 5) of the [14] in combination with a deidentification procedure. The OMOP-CDM is maintained by the Observational Health Data Sciences and Informatics network, which provides tools to facilitate data analysis. This study was approved by the Ajou University Hospital Institutional Review Board (AJIRB-MED-MDB-21-151), and the requirement for informed consent was waived owing to the deidentification.

### Calculation of RDoC Scores Using Narrative Clinical Text

**Figure 1:**
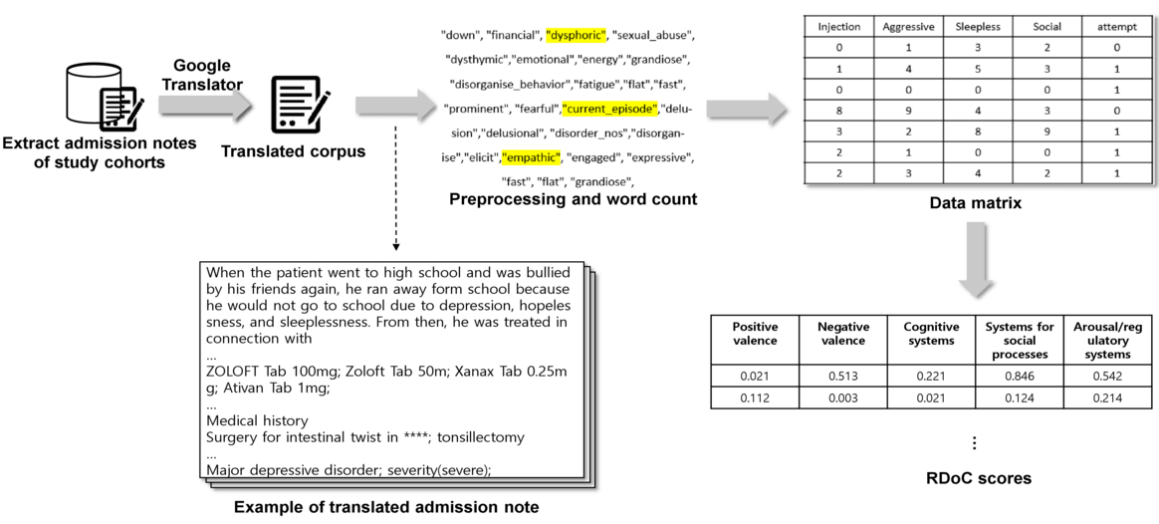
Overall workflow of extracting research domain criteria scores using a natural language processing pipeline. Admission notes were extracted from the electronic health records of the patients with diagnosis (ie, depression, schizophrenia, and bipolar disorder) and chief complaints such as suicide attempts, suicide planning, and suicidal ideation. Admission notes were translated into English by Google translator. After preprocessing, the score was calculated using the count of the predefined research domain critieria terms that appeared in the document. RDoC: research domain criteria.

McCoy et al [11] previously described a method for estimating RDoC scores from narrative text. In summary, the method evaluates a document using a predetermined set of terms belonging to a given research domain. This list of terms was developed through a group of clinical professionals, including the NIMH RDoC working group. The score can be calculated using a bag of words of the corpus and the count of predefined RDoC terms that appear in the document. For instance, if 10 terms comprise a predefined list and 2 appear in a document, the note would be assigned a score of 2/10 (20%). The list of terms predetermined by the NIMH RDoC working group is publicly available on the internet [11]. The patient admission notes in this study were written in English and Korean. The source texts were 33% in English and 67% in Korean, which were similar for each patient. However, important medical entities such as chief complaints, medical histories, medication prescriptions, and any other important descriptions that directly indicated the patients’ status are represented in English. Moreover, to minimize data loss in the corpus, we systematically translated the corpus into English that was generated by the googleLanguageR package of the R programming language (version 3.6.2) [15]. As a result, we were able to derive RDoC scores from the documents, regardless of the language, as shown in Figure 1.

### Study Design and Analysis

We conducted a retrospective cohort study to explore the differences in the RDoC domains extracted by NLP among patients with depression, schizophrenia, and bipolar disorder who were hospitalized for suicide attempts. We also investigated how these domains were associated with the length of hospital stay and compared them for each disorder. Baseline demographic and clinical data are expressed as numbers (%) for categorical variables and means (SDs) for continuous variables. Differences between psychiatric disorders were compared using analysis of variance (ANOVA) for continuous variables and Cochran Q tests for categorical variables. Post hoc analysis of the RDoC domains was conducted using independent two-sample *t* tests. Linear regression modeling with adjustments for the sex, age, and CCI was used to analyze five domains (positive valence, negative valence, cognitive systems, systems for social processes, and arousal/regulatory systems) of the RDoC in different sociodemographic profiles. For each psychiatric disorder, a multiple logistic regression model analyzing the sociodemographic variables and RDoC domains was used to identify the factors associated with the length of hospital stay. For a secondary analysis, a Cox regression model with adjustments for demographics and categorical diagnosis was used to identify each domain associated with the length of hospital stay without controlling the other four domains. As the Mental Health Promotion and Welfare Act in South Korea defines involuntary psychiatric admission within 3 days [16], hospitalization for more than 3 days indicates serious psychiatric problems. Owing to this policy, the distribution of the length of stay was also divided into less than 3 days and more than 3 days. For these reasons, we defined the length of stay as less than3 days or more than 3 days. All analyses were performed using the R programming language (version 3.6.2, R Foundation for Statistical Computing) and the open-source R packages.

## Results

The demographic and clinical characteristics of 732 admissions for 571 participants are shown in Table 1. No significant differences were observed in the age, length of stay, CCI, sex, and medical history between the three psychiatric disorder groups. Table 2 shows that significant differences are observed in the negative valence, cognitive systems, systems for social processes, and arousal/regulatory systems domains in more than two of the three psychiatric disorder groups. In the post hoc analysis of the RDoC domains in the three psychiatric disorders, negative valence was the highest in the depression group (*P*<.001), whereas cognitive systems were significantly higher in the schizophrenia group than in the depression group (*P*=.004) and in the bipolar disorder group than in the depression group (*P*<.001). Like cognitive systems, systems for social processes were significantly higher in the schizophrenia group and the bipolar disorder group than in the depression group (*P*<.001). Arousal/regulatory systems were significantly higher in the depression (*P*=.004) and bipolar disorder (*P*=.04) groups than in the schizophrenia group. Furthermore, the RDoC domains differed in their associations with sociodemographic variables given in Multimedia Appendix 1. Age was significantly associated with the RDoC domains. Patients with increased levels of arousal/regulatory systems were older, whereas patients with more systems for social processes were younger. Being male was also associated with increased levels of positive valence. On the other hand, CCI was not associated with any RDoC domain.

**Table 1:** Baseline characteristics of the patient groups (N=571).^a^

Patient characteristic			Depression (n=465)		Schizophrenia (n=73)		Bipolar disorder (n=33)		*P* value
Age (years), mean (SD)			36.4 (18.3)		31.3 (15.3)		34.8 (14.7)		.11
Length of stay, mean (SD)			8.8 (9.5)		9.9 (13.2)		6.4 (5)		.89
Charlson Comorbidity Index, mean (SD)			0.3 (0.9)		0.1 (0.4)		0.1 (0.4)		.44
Sex: female, n (%)			293 (63)		39 (53.4)		22 (66.7)		.25
**Medical history, n (%)**									
	Hypertensive disorder	62 (13.3)		5 (6.8)		4 (12.1)		.30	
	Diabetes mellitus	24 (5.2)		2 (2.7)		0 (0)		.28	
	Ischemic stroke	4 (1.5)		0 (0)		0 (0)		.45	
	Renal impairment	10 (2.2)		1 (1.4)		1 (3)		.85	
	Pneumonia	21 (4.5)		2 (2.7)		1 (3)		.74	

^a^ANOVA for continuous variables and Cochran Q tests for categorical variables were performed.

**Table 2:** Comparisons of research domain criteria scores of patients in the three psychiatric disorders (N=571).^a^

Characteristic			Depression (n=465)	Schizophrenia (n=73)		Bipolar disorder (n=33)		*P* value	Post hoc (*P* value)
**Research domain criteria score, mean (SD)**									
	Positive valence	0.112 (0.048)		0.108 (0.045)	0.098 (0.036)		.21		—^b^
	Negative valence	0.146 (0.052)		0.099 (0.041)	0.100 (0.037)		<.001		Depression > schizophrenia (<.001) depression > bipolar disorder (<.001)
	Cognitive systems	0.189 (0.096)		0.156 (0.092)	0.189 (0.096)		<.001		Schizophrenia > depression (.004) bipolar disorder > depression (<.001)
	Systems for social processes	0.112 (0.081)		0.168 (0.091)	0.176 (0.099)		<.001		Schizophrenia > depression (<.001) bipolar disorder > depression (<.001)
	Arousal/regulatory systems	0.101 (0.059)		0.080 (0.051)	0.102 (0.044)		.02		Depression > schizophrenia (.004) bipolar disorder > schizophrenia (.04)

^a^Data were analyzed by ANOVA followed by independent two-sample *t* tests during post hoc analysis.

^b^Not applicable.

Next, we examined the association between the RDoC domains extracted from the admission notes and length of hospital stay. Table 3 summarizes the results of each psychiatric disorder group. In the patient group with depression, patients who scored high in negative valence were at an increased risk of a longer length of stay (odds ratio [OR] 1.058, 95% CI 1.006-1.114). In the patient group with schizophrenia, the RDoC domains were not associated with the length of stay. In the patient group with bipolar disorder, patients who scored high in the arousal/regulatory systems were at a decreased risk of a longer length of stay (OR 0.537, 95% CI 0.285-0.815).

In the secondary analysis, compared to the primary analysis, which considered 3-day hiccups and other domains, the depression group similarly showed significant association with negative valence (Table S2 in Multimedia Appendix 1). Unlike in the primary analysis, a significant association was shown with the arousal/regulatory systems of the depression group. In the schizophrenia group, there were no significant associations as in the primary analysis. In the bipolar disorder group, unlike primary analysis, significant association was shown with positive valence.

**Table 3:** Regression model results of research domain criteria scores and length of hospital stay (N=732).

Variables		Depression (n=612)		Schizophrenia (n=83)		Bipolar disorder (n=37)	
		OR^a^ (95% CI)	*P* value	OR (95% CI)	*P* value	OR (95% CI)	*P* value
**Research domain criteria scores**							
	Positive valence	0.975 (0.935-1.018)	.25	1.016 (0.897-1.157)	.81	1.297 (0.938-1.973)	.15
	Negative valence	1.058 (1.006-1.114)	.03	1.090 (0.933-1.279)	.28	1.290 (0.967-1.860)	.11
	Cognitive systems	1.01 (0.957-1.048)	.96	1.087 (0.952-1.252)	.22	1.145 (0.887-1.561)	.33
	Systems for social processes	1.010 (0.966-1.058)	.66	0.959 (0.861-1.068)	.44	1.079 (0.881-1.361)	.47
	Arousal/regulatory systems	0.957 (0.906-1.011)	.11	0.901 (0.759-1.057)	.21	0.537 (0.285-0.815)	.02
**Sociodemographic features**							
	Age	1.000 (0.989-1.012)	.95	1.010 (0.975-1.051)	.59	1.020 (0.936-1.125)	.66
	Charlson Comorbidity Index	0.996 (0.789-1.295)	.98	1.391 (0.259-24.352)	.75	0.057 (0.000-1.924)	.49
	Sex	0.943 (0.634-1.394)	.77	1.246 (0.419-3.708)	.69	0.402 (0.014-5.742)	.52

^a^OR: odds ratio.

## Discussion

### Principal Findings

In this study, we identified statistically significant differences in the RDoC scores among psychiatric disorders and showed significant associations between the RDoC scores and length of stay for depression and bipolar disorder. The association between suicide and RDoC domains has been reported [17], but very few studies have analyzed how this relationship differs from disorder to disorder. In this regard, the present study investigated whether the RDoC scores derived by NLP differed for each disorder and whether they were related to clinical outcomes such as the length of hospital stay.

The depression group showed the highest negative valence scores among the disorder groups. Previous studies comparing the distribution of domains by diagnosis also showed differences in the negative valence scores for depression and bipolar disorder, and depression and schizophrenia [18]. Our findings not only confirm the previously established association between negative valence and suicide [19] but also suggest that negative valence is particularly associated with depression. Conversely, the schizophrenia and bipolar disorder groups scored higher than the depression group in the cognitive systems and systems for social processes, and no significant difference was found in the scores between the two groups. Several studies have suggested a significant overlap between schizophrenia and bipolar disorder [20], and similarities between these two disorders are prominent in cognitive and social functions [21,22]. Our results are in line with previous results and provide additional information that cognition and social function are important factors in suicide cases involving schizophrenia and bipolar disorder. In the arousal/regulatory systems, the bipolar disorder group scored significantly higher than the schizophrenia group, whereas no significant difference was found in the scores between the depression and bipolar disorder groups. Significant similarities in sleep features representing the arousal/regulatory systems between depression and bipolar disorder have been reported [23]. However, as schizophrenia and bipolar disorder are highly related to sleep disorders [24], it remains unclear whether the scores of the schizophrenia group in the arousal/regulatory systems are more significant than those of the bipolar disorder group.

In the current study, we found that negative valence scores were associated with a longer length of stay in the depression group (OR 1.058, 95% CI 1.006-1.114). The association found between negative valence and depression as well as suicide is consistent with previous findings [19,25]. In the schizophrenia group, no significant relationship was found between the RDoC domain and length of stay, whereas scores in the arousal/regulatory systems were associated with a shorter length of stay in the bipolar disorder group (OR 0.537, 95% CI 0.285-0.815). Contrary to our findings regarding bipolar disorder, a previous study has reported that higher arousal domain scores were associated with a longer length of stay for bipolar disorder [11]. However, sleep disturbance varies with the bipolar disorder phase [26]. These findings show that further consideration of the bipolar disorder phase is needed in interpreting the arousal domain scores with respect to bipolar disorder. Although previous studies found a significant association between the RDoC domain and length of hospital stay, some inconsistencies in prior significant relationships have been identified. For example, one study reported that a positive domain was associated with a shorter stay; however, another showed that a positive domain was associated with a longer stay [11,27]. Thus, the relationship may vary depending on the specific cohort. The association between the length of stay and RDoC domain is unclear for schizophrenia and differs from previous studies with respect to bipolar disorder because our work not only had a cohort different from that of previous studies but also analyzed the relationship between the length of stay and specific disorders.

Our findings show significant associations between the RDoC domains and length of hospital stay for depression and bipolar disorder. This result is consistent with those in existing literature reporting that the estimated RDoC domain scores were associated with the length of stay [27]. Moreover, significant differences and trends in the RDoC domains among depression, schizophrenia, and bipolar disorder were demonstrated. These findings are consistent with those of previous studies on the relationship between negative valence scores and major depressive disorders [25]. Recent publications suggest that cognitive and social functioning factors were observed in schizophrenia and bipolar disorder but not in depression, which is consistent with our findings [28]. On the other hand, using NLP to calculate the RDoC scores is more useful than using structural data alone [11]. For example, the RDoC of the cognitive domain extracted by NLP facilitated stratification of risk for dementia [29]. Our findings further validate of the usefulness and robustness of the RDoC scoring system, which identifies important clinical features in clinical notes. Furthermore, with this validated RDoC NLP tool, our study was conducted by integrating bilingual clinical notes into RDoC domains. Although prior research relied primarily on clinical notes written in English, our results show that the use of RDoC domains through NLP is appropriate for clinical notes that are not in English.

### Limitations

Our study has a few limitations. First, even though we extracted database records of suicidal patients from 2010 to 2020, we could identify only 732 psychiatric admissions in 571 patients. To validate our findings more accurately, a large data set is required. Second, our study has analyzed the conditions most highly related to suicide, but other suicide-related conditions, such as substance use disorders and personality disorders, have not been considered. Because substance use disorders and personality disorders frequently coexist with depression, schizophrenia, and bipolar disorder [30], it was difficult to distinguish between the disorders. Third, the accuracy of the translation of Korean text into English was not evaluated because this was beyond the scope of this study. The accuracy and effectiveness of using clinical NLP algorithms on multilingual clinical documents should also be investigated and validated in future.

### Conclusions

Our study showed that the estimates of dimensional psychopathology derived from NLP are associated with the length of hospital stay in suicidal patients with depression or bipolar disorder and vary significantly among suicidal patients with depression, schizophrenia, and bipolar disorder. Therefore, our findings suggest that more attention might be paid to negative valence for depression and arousal/regulatory systems for bipolar disorder in relation to suicide. Additionally, our results may increase the understanding of the differences in dimensional psychopathology among suicidal patients with depression, schizophrenia, and bipolar disorder. We hope that further investigations will clarify the differences in the RDoC scores of suicidal patients and associations between the RDoC scores of suicidal patients and clinical outcomes.

## Appendix

Multimedia Appendix 1 Association between sociodemographic features and research domain criteria scores.

## Abbreviations

ANOVA: analysis of variance
CCI: Charlson comorbidity index
CDM: common data model
DSM: Diagnostic and Statistical Manual of Mental Disorders
NIMH: National Institute of Mental Health
NLP: natural language processing
OMOP: Observational Medical Outcomes Partnership
OR: odds ratio
RDoC: research domain criteria
