# Supplementary material for: Characteristics of Dimensional Psychopathology in Suicidal Patients With Major Psychiatric Disorders and Its Association With the Length of Hospital Stay: Algorithm Validation Study
Source: JMIR Ment Health. 2021 Sep 3;8(9):e30827. doi: 10.2196/30827 (PMC8449292; doi:10.2196/30827)
Supplement: Multimedia Appendix 1 [file mental_v8i9e30827_app1.docx]

**Table S1. Association between sociodemographic features and Research Domain Criteria scoring**

|  | Positive valence  β (95% CI)^a^ | Negative valence  β (95% CI)^a^ | Cognitive systems  β (95% CI)^a^ | Systems for  social processes  β (95% CI)^a^ | Arousal/  regulatory systems  β (95% CI)^a^ |
| --- | --- | --- | --- | --- | --- |
|  |  |  |  |  |  |
| Age | -8.434 (-41.362 to 24.495) | 14.747 (-20.086 to 49.580) | 11.532 (-24.391 to 47.454) | -34.727^*^ (-67.862 to -1.593) | 72.359^*^ (31.123 to 113.594) |
| Charlson Comorbidity Index | -0.482 (-1.999 to 1.035) | 0.291 (-1.314 to 1.895) | -0.220 (-1.875 to 1.435) | -0.667 (-2.193 to 0.860) | 1.451 (-0.448 to 3.351) |
| Sex, female | -6.361^*^ (-10.381 to -2.417) | 1.061 (-3.101 to 5.246) | -2.220 (-6.529 to 2.066) | 0.840 (-3.110 to 4.817) | 2.122 (-2.800 to 7.088) |

*^a^CI, confidence interval*

*^*^P < 0.05*

Table S2. Cox regression models on length of stay via each of domain adjusting for demographics and categorical diagnosis

| Domain | HR^a^ | 95% CI^b^ | P value |
| --- | --- | --- | --- |
| Depression | | | |
| Positive valence | 1.015 | 0.999–1.031 | 0.076 |
| Negative valence | 1.019 | 1.002–1.036 | 0.026^*^ |
| Cognitive systems | 1.005 | 0.996–1.014 | 0.241 |
| Systems for social processes | 1.008 | 0.999–1.018 | 0.097 |
| Arousal/regulatory systems | 1.021 | 1.006–1.036 | 0.005^*^ |
| Schizophrenia | | | |
| Positive valence | 0.999 | 0.946–1.056 | 0.976 |
| Negative valence | 0.976 | 0.916–1.040 | 0.458 |
| Cognitive systems | 0.996 | 0.970–1.023 | 0.784 |
| Systems for social processes | 0.995 | 0.970–1.020 | 0.682 |
| Arousal/regulatory systems | 0.991 | 0.947–1.039 | 0.725 |
| Bipolar disorder | | | |
| Positive valence | 0.879 | 0.788–0.981 | 0.021^*^ |
| Negative valence | 0.982 | 0.896–1.077 | 0.699 |
| Cognitive systems | 1.006 | 0.971–1.044 | 0.728 |
| Systems for social processes | 0.999 | 0.969–1.031 | 0.995 |
| Arousal/regulatory systems | 1.086 | 0.989–1.192 | 0.083 |

*^a^HR, hazard ratio*

*^b^CI, Confidence inreval*

*^*^P < 0.05*

Table S3. Regression model of Research Domain Criteria scoring and hospital length of stay according to the time period.

| Domain | OR^a^ | 95% CI^b^ | P value |
| --- | --- | --- | --- |
| Before May 2013 | | | |
| Positive valence | 0.915 | 0.857–0.976 | 0.007^*^ |
| Negative valence | 1.074 | 1.001–1.156 | 0.051 |
| Cognitive systems | 1.016 | 0.953–1.084 | 0.622 |
| Systems for social processes | 1.019 | 0.957–1.088 | 0.570 |
| Arousal/regulatory systems | 0.934 | 0.860–1.013 | 0.102 |
| After May 2013 | | | |
| Positive valence | 1.038 | 0.986–1.095 | 0.156 |
| Negative valence | 1.067 | 1.008–1.131 | 0.026^*^ |
| Cognitive systems | 1.018 | 0.963–1.078 | 0.529 |
| Systems for social processes | 0.995 | 0.945–1.047 | 0.847 |
| Arousal/regulatory systems | 0.939 | 0.879–1.002 | 0.058 |

*^a^OR, odds ratio*

*^b^CI, Confidence interval*

*^*^P < 0.05*
